# Predictive Value of Clinicopathological Factors to Guide Post-Operative Radiotherapy in Completely Resected pN2-Stage III Non-Small Cell Lung Cancer

**DOI:** 10.3390/diagnostics13193095

**Published:** 2023-09-29

**Authors:** Ju-Chun Chien, Yu-Chang Hu, Yi-Ju Tsai, Yu-Ting Chien, I-Jung Feng, Yow-Ling Shiue

**Affiliations:** 1 Department of Radiation Oncology, Kaohsiung Veterans General Hospital, Kaohsiung 81362, Taiwan; 2 Institute of Biomedical Sciences, National Sun Yat-sen University, Kaohsiung 80424, Taiwan; 3 Department of Medical Education and Research, Kaohsiung Veterans General Hospital, Kaohsiung 81362, Taiwan; 4 School of Post Baccalaureate Chinese Medicine, China Medical University, Taichung 404333, Taiwan; 5 Institute of Precision Medicine, National Sun Yat-sen University, Kaohsiung 80424, Taiwan

**Keywords:** non-small cell lung cancer, post-operative radiotherapy, predicting factors

## Abstract

**Introduction:** With the evolution of radiotherapy techniques and a better understanding of clinicopathological factors, we aimed to evaluate the treatment effect of post-operative radiotherapy (PORT) and associated predictive factors in patients with completely resected pN2 stage III non-small cell lung cancer (R0 pN2-stage III NSCLC). **Material and Method:** The cancer registration database of a single medical center was searched for R0 pN2-stage III NSCLC. Clinicopathological factors and information about post-operative therapies, including PORT and adjuvant systemic treatment, were retrospectively collected and analyzed. The Kaplan-Meier method and a Cox regression model were applied for time-to-event analysis, with disease-free survival (DFS) being the primary outcome. **Results:** From 2010 to 2021, 82 R0 pN2-stage III NSCLC patients were evaluated, with 70.1% of tumors harboring epidermal growth factor receptor mutations (EGFR mut.). PORT was performed in 73.2% of cases, and the median dose was 54 Gy. After a median follow-up of 42 months, the 3-year DFS and overall survival (OS) rates were 40.6% and 77.3%, respectively. Distant metastasis (DM) was the main failure pattern. In the overall cohort, DFS was improved with PORT (3-year DFS: 44.9% vs. 29.8%; HR: 0.552, *p* = 0.045). Positive predictive factors for PORT benefit, including EGFR mut., negative extranodal extension, positive lymphovascular invasion, 1–3 positive lymph nodes, and a positive-to-dissected lymph node ratio ≤0.22, were recognized. OS improvement was also observed in subgroups with less lymph node burden. **Conclusions:** For R0 pN2-stage III NSCLC, PORT prolongs DFS and OS in selected patients. Further studies on predictive factors and the development of nomograms guiding the application of PORT are highly warranted, aiming to enhance the personalization of lung cancer treatment.

## 1. Introduction

Lung cancer is the leading cause of cancer mortality and is ranked second in incidence among malignancies worldwide [1]. Based on histology, it is categorized into non-small cell lung cancer (NSCLC) and small cell lung cancer, with the former accounting for 81% of all lung cancer diagnoses. For localized early-stage NSCLC, radical surgery is the backbone of cancer treatment. The addition of adjuvant therapy is considered if a margin-negative (R0) resection is not achieved or if pathologically proven advanced disease is observed. Systemic treatment options, such as platinum-based chemotherapy (CT), epidermal growth factor receptor tyrosine kinase inhibitors (EGFR-TKIs), or immune checkpoint inhibitors (ICIs), are increasingly used post-operatively given their well-established disease-free survival (DFS) benefit [2,3,4]. On the other hand, the role of post-operative radiotherapy (PORT) in NSCLC remains under debate.

Previous review articles have suggested that the effectiveness of PORT in R0 NSCLC was underestimated due to diminished benefits and relatively high radiation toxicity with the inclusion of patients with early-stage disease and the application of old radiotherapy techniques in early studies [5,6]. Therefore, recent studies have focused on patients with mediastinal lymph node involvement (pN2-stage III) and have applied modern radiotherapy techniques. In two recent large randomized control studies, the EORTC 22055/Lung ART trial and the PORT-C trial, both revealed no disease-free survival difference with the addition of PORT to R0 pN2-stage III NSCLC, despite a decreased mediastinal relapse [7,8]. The Lung ART trial reported high radiation toxicity with 16 cases in the PORT group dying from cardiopulmonary disease, compared to 2 in the control group [7]. This might be attributed to the fact that 89% of PORT was performed using the relatively outdated 3D conformal radiotherapy technique. On the other hand, radiotherapy was administered using intensity-modulated radiation therapy (IMRT) to 89.3% of cases in the PORT-C trial, and the study was conducted in East Asia, where epidermal growth factor receptor mutations (EGFR muts.) are more common in NSCLC [8,9,10]. A high protocol violation rate might influence the results of intention-to-treat analysis in the PORT-C trial, while superior DFS with PORT was observed in per-protocol analysis (HR: 0.75, *p* = 0.05) and in an exploratory analysis with stratification based on the number of dissected lymph nodes (DLNs) and positive lymph nodes (PLNs) (HR: 0.75, *p* = 0.04) [8].

Traditionally, radiotherapy was considered a localized treatment modality. By eradicating cancer cells within the mediastinal field, PORT might decrease locoregional recurrence and should prevent subsequent distal failure and cancer death. However, in the two large randomized control trials, distal metastasis was the main failure pattern in both the PORT and control groups, indicating that a poor prognostic factor might not necessarily be a good predictive factor for PORT if it leads to excessive distal failure risk [7,8]. Surgical margin status is a well-recognized parameter used to evaluate local recurrence risk. Nevertheless, only approximately 30% of the recruited cases in the Lung ART trial met the criteria for R0 resection in accordance with the International Association for the Study of Lung Cancer (IASLC), where not only the integrity of all margins, but also a predefined systematic nodal dissection, the absence of extranodal extension (ENE) of the positive node removed separately and of the highest mediastinal basin, and negativity of the highest mediastinal node removed are required, limiting its clinical applicability [7,11]. 

According to the latest National Comprehensive Cancer Network (NCCN) guideline, PORT is listed as an optional treatment for R0 pN2-stage III NSCLC with high-risk factors, such as ENE, multi-station involvement, inadequate dissected lymph nodes, and those not completing adjuvant systemic therapy. However, there was limited supporting evidence for the influence of these factors on the PORT effect. Considering the poor clinical application of the IASLC criteria for margin status and the lack of evidence to guide PORT beyond pN2-stage III NSCLC, we aimed to evaluate the predictive value of clinicopathological factors and to provide further guidance for the decision to administer PORT.

## 2. Materials and Methods

### 2.1. Study Cohort and Data Collection

**Figure 1:**
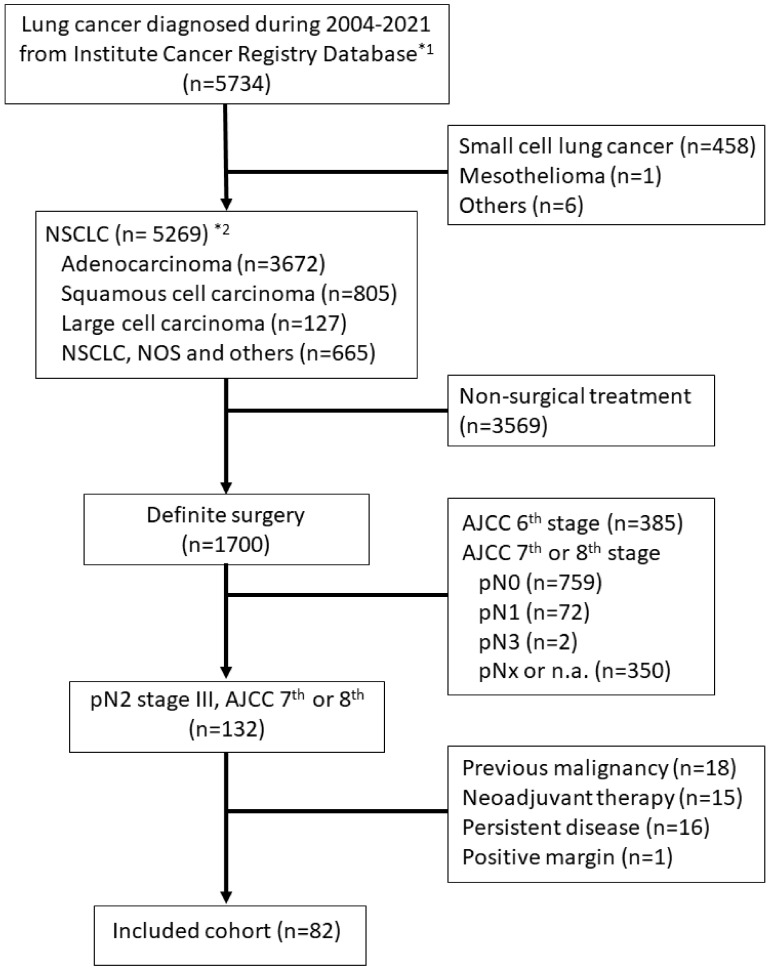
Flow diagram for case inclusion in the study cohort. *^1^ The Institute Cancer Registry Database contains all malignant disease records diagnosed in the facility, dating back from 2004 to 2021, the time of the data request. *^2^ Non-small cell lung cancer was classified according to the definition of the World Health Organization (WHO)/International Association for the Study of Lung Cancer (IASLC), and patients were identified from the database using the ICD-O-3 code. Abbreviations: NSCLC, non-small cell lung cancer; NOS, not otherwise specified; AJCC, American Joint Committee on Cancer, Cancer staging manual.

The cancer registry database of a medical center, including all NSCLC cases diagnosed in the facility, was retrospectively screened for pathologically proven pN2-stage III NSCLC patients, staged according to the 7th or 8th editions of the American Joint Committee on Cancer (AJCC) staging manual. Patients should have received definite surgery with procedures, such as lobectomy, segmentectomy, or wedge resection, along with lymph node dissection or sampling. Complete surgical resection, defined as no tumor involvement of the resection margins and no positive cytology of pleural or pericardial effusion, was required. Patients who received neo-adjuvant treatment of any type prior to definite surgery and those with a positive surgical margin, lymph node involvement in the contralateral or supraclavicular regions (N3), known distant metastasis (M1), or history of previous malignancies were excluded. The flow diagram of case inclusion is presented in Figure 1.

Baseline characteristics, treatment course, and histopathological factors, including but not limited to EGFR mut. status, ENE, PLNs, DLNs, and positive-to-dissected lymph node ratio (PD ratio), which was defined as the value of positive lymph node number divided by dissected lymph node number, were recorded. Recurrent and survival data were retrospectively collected from medical charts, hospital cancer registry records, and the National Death Registry. The study was approved by the institutional review board of our facility.

### 2.2. Definition of Endpoints

The primary endpoint was disease-free survival (DFS), defined as survival without evidence of disease. The coding of recurrence or metastasis was mainly based on medical records. For events not specified in the medical chart, an experienced radiation oncologist with expertise in thoracic malignancy, who was not aware of the study hypothesis and allocation at the time, was consulted. Lesions located in the mediastinum or around the hilum of the ipsilateral lung were coded as locoregional recurrence (LRR), while thoracic lesions beyond the above-mentioned area or extra-thoracic lesions were coded as distant metastases (DMs). Regarding cause of death, a death reported from the National Death Registry without a specific cause of death was coded as a non-cancer death, representing an event in overall survival (OS) but censored in disease-specific survival (DSS) analysis. All endpoints were evaluated in time-to-event analysis, starting from the date of definite surgery.

### 2.3. Statistical Analysis

The software Statistical Product and Service Solutions Statistics (SPSS statistics, 22nd) was used for data analysis. The distribution of the use of PORT based on baseline characteristics was compared using the Pearson’s chi-square test for possible imbalance. DFS, LRR, DM, and survival outcomes were analyzed using the Kaplan-Meier method. The Cox regression model was used to recognize possible prognostic factors. Factors achieving a *p*-value less than 0.1 in univariate analysis were kept for multivariate tests. A *p*-value less than 0.05 was considered statistically significant. 

To evaluate the treatment effect of PORT in each subgroup, hazard ratios (HRs) were calculated using the Cox regression model with stratification according to clinicopathological factors. The HRs were adjusted for prognostic factors and factors with an uneven distribution in multivariate analysis to prevent possible bias.

## 3. Results

From 2010 to 2021, 82 consecutive patients with R0 pN2-stage III NSCLC were identified, and 89.0% were diagnosed with adenocarcinoma. Fifty-five (67.1%) patients had ^18^F-FDG PET/CT exams for pre-operative staging workup. After surgery, only one patient received an adjuvant EGFR-TKI, and 73.2% of the cases completed at least 4 courses of platinum-based chemotherapy. 

Regarding radiotherapy, 60 patients underwent PORT, among which 84.5% received volumetric modulated arc therapy (VMAT). The patients were immobilized with a thermoplastic cast or wing board with free breathing since the respiratory motion was less significant in the PORT treatment field, namely, the mediastinal lymph node basins. Most patients (71.7%) received 50–54 Gy in 25–30 fractions, and boostirradiation to 58–60 Gy was given for some patients with ENE. The dose constraints and the mean value of planned dosimetry parameters were V20 < 30% (mean: 19.7%) and V30 < 20% (mean: 13.8%) for the bilateral lung, respectively, with a mean heart dose <20 Gy (mean: 11.2 Gy). The distribution of PORT according to baseline characteristics is presented in Table 1. A statistically non-significant trend of imbalance was observed with a greater portion of patients completing 4 cycles of platinum-based chemotherapy or presenting with lymphovascular invasion (LVSI) receiving PORT.

### 3.1. Treatment Outcomes and Prognostic Factors

After a median follow-up of 42 months (IQR: 29–62 months), 11 locoregional recurrences, 53 distant metastases, and 34 mortality were recorded. The overall 3-year DFS was 40.6% (95% CI: 29.6–51.6%; median: 22 months). The 3-year LRR and DM risks were 13.7% (95% CI: 5.7–21.7%) and 57.9% (95% CI: 46.7–69.1%), respectively. The 3-year DSS was 80.7% (95% CI: 71.1–90.3%), and the 3-year OS was 77.3% (95% CI: 67.3–87.3%).

In terms of prognostic factors, elevated pre-operative tumor markers and not receiving PORT were associated with a worse DFS in multivariate analysis (Table 2). On the other hand, PORT might reduce LRR (HR: 0.092, *p* < 0.001, 95% CI: 0.024–0.351), and a PD ratio >0.22 increased the risk of DM (HR: 1.846, *p* = 0.027, 95% CI: 1.072–3.179; Table 2). No significant prognostic factor for DSS or OS was found in our cohort.

### 3.2. Disease-Free Survival Benefit of PORT and Predicting Factors

**Figure 2:**
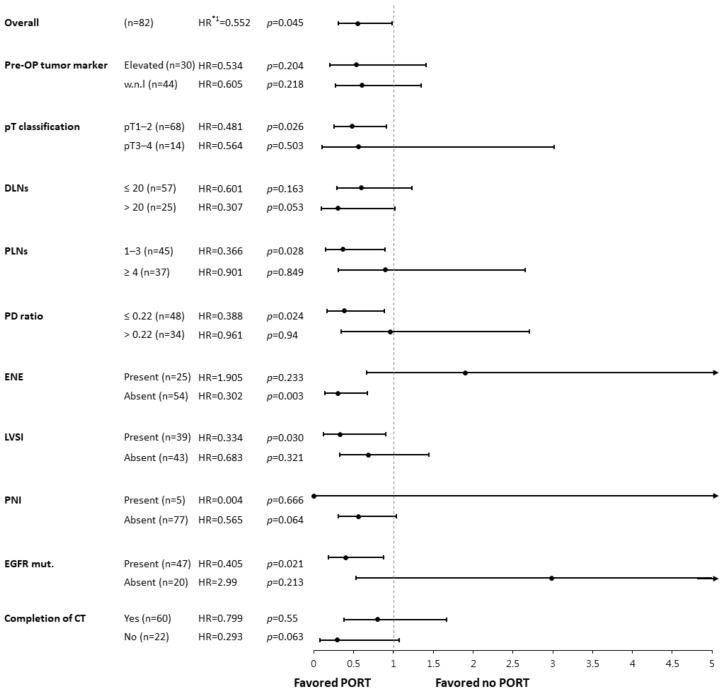
Forest plot of the post-operative radiotherapy effect on disease-free survival among subgroups. *^1^ The hazard ratios (HRs) displayed above, stratified by clinicopathological factors, were adjusted for pre-OP tumor markers and PD ratio using a Cox regression model. The forest plot was then constructed using the software Comprehensive Meta-Analysis (CMA). Abbreviations: Pre-OP, pre-operative; DLNs, dissected lymph nodes; PLNs, positive lymph nodes; PD ratio, positive to dissected lymph node ratio; ENE, extranodal extension; LVSI, lymph-vascular space invasion; PNI, peri-neural invasion; EGFR mut., epidermal growth factor receptor mutant; CT, chemotherapy; PORT, post-operative radiotherapy; HR, hazard ratio.

When stratified by PORT, the 3-year DFS was 44.9% vs. 29.8% (95% CI: 32.0–57.8% vs. 9.8–49.8%; median: 27.0 vs. 17.0 months), and the 3-year OS was 76.3% vs. 80.1% (95% CI: 64.5–88.1% vs. 52.5–97.7%; median: 79.0 vs. 54.0 months) for those with vs. without PORT, respectively. Since the DFS benefit of PORT was observed in our cohort but not in many previous studies, we conducted a subgroup analysis to evaluate possible predictive factors. The effect of PORT on DFS is presented in Figure 2 using HRs adjusted for prognostic factors, including pre-operative tumor markers and the PD ratio. The benefit of PORT was more evident for pT1–2 tumors, 1–3 PLNs, a PD ratio ≤0.22, absence of ENE, presenting with LVSI, and EGFR mutation compared to their counterparts. Although the difference between pT classification was likely caused by the contrasting case numbers, other factors were considered possible predictive factors for PORT in R0 pN2-stage III NSCLC patients.

### 3.3. Possible Survival Benefits from PORT in Subgroups

**Figure 3:**
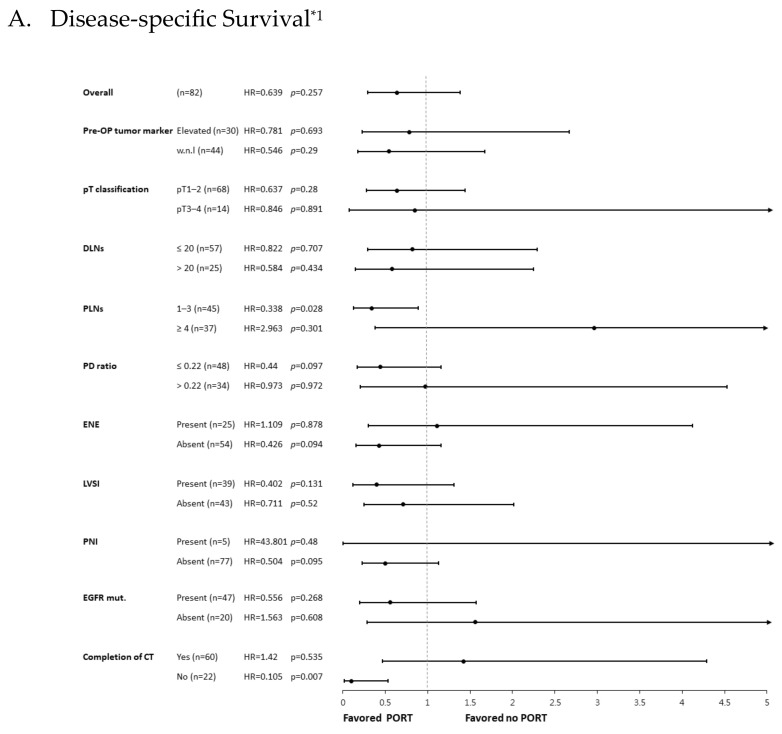

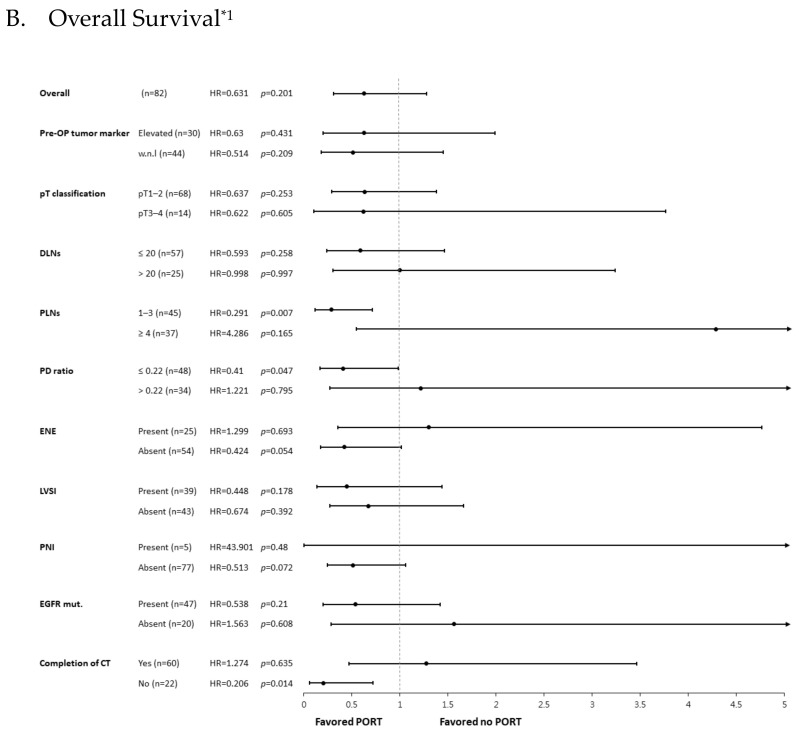
Forest plot of the post-operative radiotherapy effect on DSS and OS among subgroups. *^1^ The hazard ratios (HRs) displayed above were not corrected in the Cox regression model, except for stratification according to the listed clinicopathological factors. The forest plot was then constructed using the software Comprehensive Meta-Analysis (CMA). Abbreviations: DSS, disease-specific survival; OS, overall survival; Pre-OP, pre-operative; DLNs, dissected lymph nodes; PLNs, positive lymph nodes; PD ratio, positive to dissected lymph node ratio; ENE, extranodal extension; LVSI, lymph-vascular space invasion; PNI, peri-neural invasion; EGFR mut., epidermal growth factor receptor mutant; CT, chemotherapy; PORT, post-operative radiotherapy; HR, hazard ratio.

Although no DSS or OS improvement was noted with PORT in the overall cohort, significant superior survival was observed in several subgroups. A better DSS was found in the subgroup of patients with 1–3 PLNs receiving PORT. Improved OS was noticed in the 1–3 PLNs and PD ratio ≤0.22 subgroups with PORT, while a similar trend was observed in patients without ENE (Figure 3). These results were compatible with the DFS analysis, indicating that the DFS benefit of PORT might translate into better DSS and OS in specific subgroups. Unexpectedly, the DSS and OS benefit of PORT was also found in those not completing 4 cycles of platinum-based chemotherapy (Figure 3).

## 4. Discussion

In this retrospective cohort study carried out in an area with a high incidence of EGFR mutation-positive NSCLC and using a modern radiotherapy technique, PORT was associated with an improved DFS rate in patients with R0 pN2-stage III NSCLC. The DFS benefit was more profound in subgroups with fewer PLNs, a lower PD ratio, the absence of ENE, and the presence of LVSI and EGFR mutation. The potential translation of the DFS improvement into superior DSS and OS was also observed in those with low lymph node burden. 

Using the study cohort developed from a single medical center database, we have achieved treatment outcomes compatible with those of recent clinical trials. In our analysis, the 3-year DFS was 44.9% with PORT and 29.8% without. Similarly, the PORT-C trial reported a 3-year DFS rate of 41% vs. 33% and 43% vs. 31% for the intention-to-treat and per-protocol analysis, respectively [8]. A slightly higher distant metastasis risk was observed, probably given that 26.8% of patients included in the study did not complete CT and the limited use of adjuvant EGFR-TKIs or ICIs in our cohort. Despite the relatively low DFS compared to that of the Lung-ART trial, the PORT-C trial and our cohort both yielded favorable 3-year OS rates of 80% and 77.3%, respectively [7,8]. The discrepancy was hypothesized to be linked to the higher incidence of EGFR-addicted tumors in the PORT-C trial in China as these tumors are associated with patients of a younger age, non-smokers, and those with a good response to EGFR-TKIs [10]. 

The EGFR mutation has been a well-known prognostic factor for superior progression-free and overall survival in NSCLC, even without EGFR-TKIs [12,13]. It is more commonly detected in adenocarcinomas of the lung in Asian, female, and non-smoking populations [14,15]. The presence of an EGFR mutation was considered a positive predictive factor for PORT in our study. The driver gene mutations were also associated with the PORT effect in a cohort study conducted in China, focusing on pN2 NSCLC with uncertain resection margins. An overall survival improvement from PORT was only observed in those with a positive driver gene mutation [16]. This might reflect the optimistic result for PORT in the PORT-C trial when compared to the Lung-ART trial. Research on the cross-reactivity between an EGFR mutation and PORT is limited, and the mechanism is not known. Hypotheses, such as less systemic hypoxia-induced radioresistance in non-smokers with EGFR mutations or an interaction within the PI3K/AKT/mTOR signaling pathway, which is shared by EGFR downstream activation and radiotherapy cytotoxicity, might warrant further research [17]. 

Several other potential predictive factors for PORT were identified in our cohort. The DFS benefit was more pronounced for factors primarily associated with a favorable prognosis, and an indistinct trend for lower distant metastasis (DM) risk was observed (Table 2). Traditionally, radiotherapy is recognized as a local treatment modality that eradicates cancer cells within the irradiated field. As distant metastasis is the main failure pattern, very high-risk pN2-stage III NSCLC patients deemed to have malignant cells beyond the mediastinum would experience limited benefits from PORT. A positive extranodal extension of the involved lymph node is one of the suggested indications for PORT in the NCCN guideline. However, the presence of a PORT benefit was only observed in the ENE-negative subgroup, not the ENE-positive subgroup, in our cohort. The same phenomenon that PORT paradoxically led to improved OS in resected pN2 NSCLC patients with a negative ENE status but not with a positive ENE status was also reported by Moretti et al. They speculated that a positive ENE status may indicate a higher risk for clinically occult distant metastasis at the time of surgery [18]. This might be supported by the association of ENE with poor distant recurrence-free survival (HR: 3.42, *p* < 0.001), and the link was even stronger than the prediction of locoregional recurrence-free survival (HR: 2.21, *p* = 0.004) [19]. On the other hand, the presence of LVSI has a more significant impact on nodal recurrence risk (5-year cumulative incidence of nodal recurrence: 22.5% vs. 8.7%, *p* < 0.001, RR: 2.6) than on distant metastasis (30.4% vs. 14.9%, *p* = 0.004, RR: 2.0) [20] and would, thus, be linked to the DFS benefit of PORT. 

While the prognostic value of PLNs and the PD ratio has been well-recognized and verified in pN2 NSCLC patients receiving PORT [21], controversy exists regarding the use of lymph node burden as a predictive factor for PORT. In serial analyses based on the Surveillance, Epidemiology, and End Results (SEER) database, Wu et al. included all resected stage III NSCLC patients and reported a significant OS improvement with PORT in the stage IIIA/pN2 and PD ratio > 1/3 subgroups [22]. Urban et al. and Wang et al., focusing on resected pN1–2 and pN2-stage IIIA NSCLC, suggested a more profound PORT survival benefit with a PD ratio >50% or PLNs > 3, respectively [23,24]. In another study evaluating a similar cohort to ours and using a more sophisticated definition of PORT, Lee et al. proposed that a PD ratio of 0.6–0.8 was the optimal indicator of PORT benefit for pN2 NSCLC patients [25]. Nonetheless, the lack of information regarding surgical margin status and radiotherapy details in the SEER database should be considered when interpreting these results. It would be useful if the analysis of PLNs and the PD ratio from recent two large randomized control trials was available. Other clinicopathological factors, including the lymph node counts of only N2 nodes, the number of stations involved, or stratification with DLNs, might warrant further evaluation for the true influence of lymph node burden [26,27,28]. 

The DSS and OS advantages of PORT were observed for subgroups with DFS improvements. This might ensure the safety of modern radiotherapy with manageable toxicity and an overall gain from PORT. For subgroups with a PD ratio ≤0.22, a superior OS was found without DSS benefit. The difference might be attributed to the fact that 6 out of the 34 mortality events were coded as non-cancer deaths with no pre-specified cause of death provided in the National Death Registration.

The findings of this study should be considered in light of some limitations. The retrospective cohort study design might cause selection bias, such as the non-significant trend of a baseline imbalance regarding LVSI and chemotherapy completion observed in our cohort. Considering LVSI as a poor prognostic factor, patients with positive LVSI were more likely to receive PORT. Nevertheless, a superior disease-free survival was still observed in the intensively treated PORT subgroup, and the PORT benefit was also evident in the LVSI-positive subgroup. On the other hand, patients not completing chemotherapy and those without PORT were more likely to have an Eastern Cooperative Oncology Group (ECOG) performance score of 1, rather than 0. This might cause an overestimation of the PORT benefit. Further prospective studies are essential to eliminate the impact of these confounding factors. The study cohort was developed from an EGFR mutant-NSCLC pandemic area with restricted use of adjuvant EGFR-TKIs and ICIs. These findings should be carefully evaluated in other geographic areas and in the context of the use of modern systemic therapy. 

## 5. Conclusions

Among R0 pN2-stage III NSCLC patients, PORT prolongs DFS and OS in selected subgroups. Patients with an EGFR mutation, the presence of LVSI, a negative ENE of an involved node, and less lymph node burden derive greater benefits from PORT. Patients who meet the criteria mentioned above should be offered the option of PORT through a shared decision-making model, given its clear benefit in locoregional control and the potential for improved survival when modern radiotherapy techniques are applied. Further research and the development of nomograms guiding the application of PORT are highly warranted, aiming to enhance the personalization of lung cancer treatment. 

## Figures and Tables

**Table 1:** Baseline Characteristics and Distribution of Post-operative Radiotherapy.

Subgroup		*n*	Port	No Port	*p* *^1^
Overall		82	73.2%	26.8%	
Age	≤60 y/o	41	75.6%	24.4%	0.618
	>60 y/o	41	70.7%	29.3%	
Sex	Male	34	70.6%	29.4%	0.657
	Female	48	75.0%	25.0%	
Laterality	Right lung	45	71.1%	28.9%	0.642
	Left lung	37	75.7%	24.3%	
Pre-OP tumor marker *^2^	Elevated	30	66.7%	33.3%	0.459
	w.n.l.	44	75%	25%	
	n.a.	8	87.5%	12.5%	
pT classification *^3^	pT1–2	68	75%	25%	0.410
	pT3–4	14	64.3%	35.7%	
Histology	Adeno.	73	71.2%	28.8%	0.327
	SCC	4	75%	25%	
	Others	5	100%	0%	
Grade	Grade 2	50	70%	30%	0.418
	Grade 3	32	78.1%	21.9%	
DLNs	≤20	57	75.4%	24.6%	0.484
	>20	25	68%	32%	
PLNs	1–3	45	66.7%	33.3%	0.143
	≥4	37	81.1%	18.9%	
PD ratio *^4^	≤0.22	48	68.8%	31.2%	0.283
	>0.22	34	79.4%	20.6%	
ENE	Present	25	68.0%	32.0%	0.736
	Absent	54	75.9%	24.1%	
	n.a.	3	66.7%	33.3%	
LVSI	Present	39	82.1%	17.9%	0.084
	Absent	43	65.1%	34.9%	
PNI	Present	5	80%	20%	0.722
	Absent	77	72.7%	27.3%	
EGFR mut.	Present	47	68.1%	31.9%	0.525
	Absent	20	80%	20%	
	n.a.	15	73.3%	26.7%	
Completion of CT *^5^	Yes	60	78.3%	21.7%	0.081
	No	22	59.1%	40.9%	

*^1^ The *p*-value of the 2-tailed Pearson’s chi-square test. *^2^ Elevated tumor marker was defined as serum CEA ≥ 5 ng/mL or SCC ≥ 1.5 ng/mL before surgery. *^3^ All pT classifications were adjusted and recorded according to AJCC 8th edition. *^4^ PD ratio was the value of the positive lymph node number divided by the dissected lymph node number. *^5^ Patients receiving at least 4 cycles of platinum-based chemotherapy were recorded as completed CT. Abbreviations: PORT, post-operative radiotherapy; y/o, year-old; Pre-OP, pre-operative; w.n.l., within normal limitation; n.a., not available; Adeno., adenocarcinoma; SCC, squamous cell carcinoma; DLNs, dissected lymph nodes; PLNs, positive lymph nodes; PD ratio, positive to dissected lymph node ratio; ENE, extranodal extension; LVSI, lymph-vascular space invasion; PNI, peri-neural invasion; EGFR mut., epidermal growth factor receptor mutant; CT, chemotherapy.

**Table 2:** Prognostic Value of Clinicopathological Factors.

		Distant Metastasis		Disease-Free Survival			
		Univariate		Univariate		Multivariate *^5^	
Factors		HR	*p*	HR	*p* *^5^	HR	*p*
Pre-OP tumor marker *^1^	Elevated	1.532	0.150	1.698	0.063	1.823	0.039
pT classification *^2^	pT3–4	0.526	0.140	0.643	0.250		
DLNs	>20	0.901	0.735	0.865	0.624		
PLNs	≥4	1.574	0.102	1.359	0.251		
PD ratio *^3^	≤0.22	1.846	0.027	1.630	0.068	1.646	0.080
ENE	+	1.418	0.224	1.299	0.347		
LVSI	+	1.361	0.272	1.269	0.386		
PNI	+	1.484	0.452	1.803	0.261		
EGFR mut.	+	1.058	0.862	1.157	0.650		
Completion of CT *^4^	+	0.695	0.227	0.736	0.292		
PORT	+	0.697	0.221	0.541	0.030	0.552	0.045

*^1^ Elevated tumor marker was defined as serum CEA ≥ 5 ng/mL or SCC ≥ 1.5 ng/mL before surgery. *^2^ All pT classifications were adjusted and recorded according to AJCC 8th edition. *^3^ PD ratio was the value of the positive lymph node number divided by the dissected lymph node number. *^4^ Patients receiving at least 4 cycles of platinum-based chemotherapy were recorded as completed CT. *^5^ Factors with *p*-value < 0.1 in the univariate analysis were kept for multivariate analysis. Abbreviations: Pre-OP, pre-operative; DLNs, dissected lymph nodes; PLNs, positive lymph nodes; PD ratio, positive to dissected lymph node ratio; ENE, extranodal extension; LVSI, lymph-vascular space invasion; PNI, peri-neural invasion; EGFR mut., epidermal growth factor receptor mutant; CT, chemotherapy; PORT, post-operative radiotherapy; HR, hazard ratio.

## Data Availability

Data are not publicly available.

## Abbreviations

NSCLC	non-small cell lung cancer
PORT	post-operative radiotherapy
EGFR mut.	epidermal growth factor receptor mutation
ENE	extranodal extension
PLNs	number of positive lymph nodes
DLNs	number of dissected lymph nodes
PD ratio	Positive-to-dissected lymph node ratio
